# Carbenoxolon Is Capable to Regulate the Mitochondrial Permeability Transition Pore Opening in Chronic Alcohol Intoxication

**DOI:** 10.3390/ijms221910249

**Published:** 2021-09-23

**Authors:** Yulia Baburina, Irina Odinokova, Olga Krestinina

**Affiliations:** Institute of Theoretical and Experimental Biophysics, Russian Academy of Sciences, Pushchino, 142290 Moscow, Russia; odinokova@rambler.ru (I.O.); ovkres@mail.ru (O.K.)

**Keywords:** carbenoxolone (Cbx), rat liver mitochondria (RLM), chronic ethanol intoxication, mitochondrial permeability transition pore (mPTP)

## Abstract

Background: carbenoxolone, which is a derivative of glyceretic acid, is actively used in pharmacology for the treatment of diseases of various etiologies. In addition, we have shown carbenoxolone as an effective inducer of mitochondrial permeability transition pore in rat brain and liver mitochondria. Methods: in the course of this work, comparative studies were carried out on the effect of carbenoxolone on the parameters of mPTP functioning in mitochondria isolated from the liver of control and alcoholic rats. Results: within the framework of this work, it was found that carbenoxolone significantly increased its effect in the liver mitochondria of rats with chronic intoxication. In particular, this was expressed in a reduction in the lag phase, a decrease in the threshold calcium concentration required to open a pore, an acceleration of high-amplitude cyclosporin-sensitive swelling of mitochondria, as well as an increase in the effect of carbenoxolone on the level of mitochondrial membrane-bound proteins. Thus, as a result of the studies carried out, it was shown that carbenoxolone is involved in the development/modulation of alcohol tolerance and dependence in rats.

## 1. Introduction

Solving the problems associated with the consequences of chronic alcohol intoxication is an urgent and important task for science all over the world. The pathological effect on the organism is very diverse and affects almost all organs and tissues; however, the liver is most damaged, which serves as a filter for toxins into which alcohol breaks down. Under the influence of alcohol, hepatocytes are damaged and killed, redox processes are disturbed, acetaldehyde accumulates, which is a toxic product during the breakdown of alcohol, inflammation and replacement of liver cells with connective tissue develops. All this leads to the occurrence of pathological conditions and, ultimately, results in the development of various dangerous diseases such as liver fibrosis, alcoholic steatohepatitis, cirrhosis and hepatocellular cancer [1,2,3].

Chronic consumption of alcohol is also associated with an increase in the level of circulation of endotoxins and pro-inflammatory cytokines affecting the liver [4]. The mechanism for increasing the sensitivity of hepatocytes to external stress is unknown, but it is believed that it includes a violation of mitochondrial functions and anti-stress protective pathways, as well as the activation of pro-apoptotic signaling pathways. At the same time, the obtained data indicate that mitochondrial dysfunction, associated with oxidative stress, as well as impaired anti-stress defense mechanisms and activation of pro-apoptotic signaling pathways underlie pathological changes in organs and tissues in chronic alcoholism [5,6].

The damaging effect of ethanol on mitochondria is also represented in the disturbance of the integrity and functioning of mitochondrial membranes and can be mediated by several factors. Among them can note a decrease of mitochondrial resistance to apoptosis inducers (Ca^2+^, ROS, pro-apoptotic proteins of the Bcl-2 family) and facilitation of permeabilization of mitochondrial membranes, in particular, by the functioning of mitochondrial permeability transition pore (mPTP) and mitochondrial outer membrane permeability (MOMP) mechanisms [7,8,9].

Moreover, our studies and the work of other groups have shown the direct involvement of mPTP in the mechanisms acting in the mitochondria in chronic alcoholism [8,10,11]. It is known that the effect of alcohol in the mitochondria of the liver triggers the opening of mPTP [7,11]. We also obtained data about the existence of a new mechanism in rat liver mitochondria (RLM) in response to degenerative changes caused by ethanol that includes changes in the functioning of mPTP, the level of key regulators of mPTP and is possibly regulated by protein kinases [11]. In this work, we continued to study the operation of this system, as well as to search for objects of influence mPTP and potential regulators of its functioning.

Alcohol is known to activate glucocorticoids, which in turn enhance the damaging influence of alcohol [12]. The effect of glucocorticoids is modulated by the activity of 11β-hydroxysteroid dehydrogenases (11β-hydroxysteroid dehydrogenase (11β-HSD). Alcohol destroys the regulation of glucocorticoids in rodents and humans [13]. Recent studies have shown that carbenoxolone, a non-selective 11β-HSD inhibitor, can reduce alcohol consumption [14]. Carbenoxolone, (Cbx,—an ester of 18-alpha-glyceretic acid), obtained from licorice and used as an anti-ulcer, anti-inflammatory, antiviral and antitumor agent, improves a person’s ability to remember and analyze information. Cbx improves brain function in healthy older adults and diabetics with mental health problems [15]. In addition, Cbx has an anticonvulsant effect, induces muscle relaxation, believed to be blocking intercellular contacts [16]. There are also the series of works showing that the effect of Cbx on RLM is reflected in an acceleration of mitochondrial swelling and a decrease in the mitochondrial membrane potential [17,18,19]. These effects were accompanied by the generation of hydrogen peroxide, an increase in oxygen uptake and oxidation of SH- and pyridine nucleotides [20]. Thus, the action of Cbx in mitochondria is associated with the functioning of the mPTP [21]. Taking into account the above, as well as our data showing that Cbx is able to take part in the modulation of mitochondrial functions [21], in this work, the effect of Cbx on the functional state of liver mitochondria, the parameters of mPTP functioning, changes in the level of mitochondrial proteins in control rats and rats subjected to chronic alcohol intoxication were studied.

## 2. Results

At first, we investigated changes in the parameters of the functioning of mPTP in the liver mitochondria isolated from control animals (Control RLM) and rats with chronic alcohol intoxication (Ethanol RLM), as well as when Cbx was added to mitochondria.

**Figure 1:**
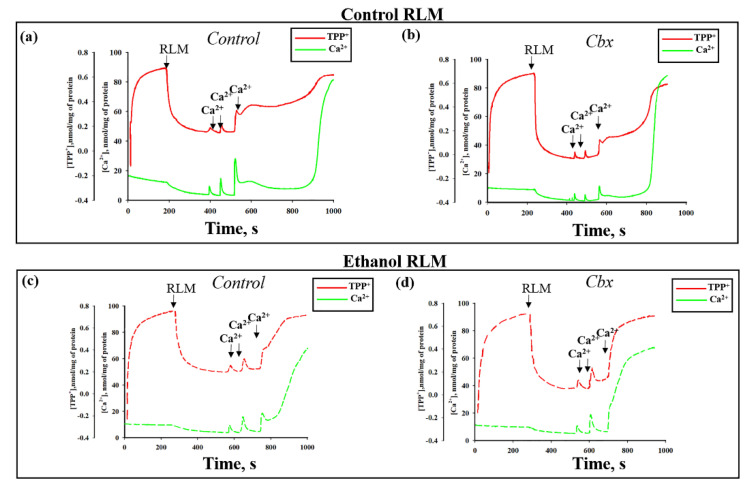
Effect of Carbenoxolone (Cbx) on the Ca^2+^ transport (green lines) and membrane potential changes (red lines) in the Control (**a**,**b**) end Ethanol (**c**,**d**) RLM. The arrows indicate where CaCl_2_ (50, 90 and 90 μM, respectively) was added to the mitochondrial suspension. RLM (1 mg/mL) were incubated in a standard medium, as described in Section 4.

**Figure 2:**
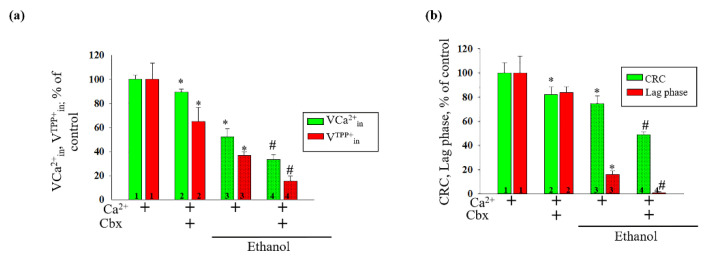
Quantitative characteristics of the effect of Cbx on the Ca^2+^-induced mPTP opening in the Control and Ethanol RLM; (**a**) quantitative analysis of V^Ca2+^_in_ and V^TPP+^_in_; (**b**) quantitative analysis of the CRC (calcium retention capacity) and of the lag phase. The values shown are the means ± SD from three independent experiments; * *p* ≤ 0.05 compared with the control value in the RLM without additions (columns 1); # *p* ≤ 0.05 compared with the control value in the Ethanol RLM without additions (dotted columns 3).

Figure 1 shows the curves of changes in the Ca^2+^ and TPP^+^ (indicating the changes in mitochondrial membrane potential, ΔΨm) flows under different experimental conditions. Pulses of Ca^2+^ were added to the mitochondria to reach a threshold Ca^2+^ concentration for the mPTP opening. The first two additions of Ca^2+^ (50 and 90 nmol per mg of protein) led in all cases to a stable accumulation of Ca^2+^ in the mitochondria followed by the recovery of ΔΨm. The threshold Ca^2+^ concentration (a calcium concentration that leads to the mPTP opening) was approximately 190 µM. In the Control RLM (Figure 1a), the third addition of Ca^2+^ (90 nmol per mg of protein) induced the mPTP opening after a long lag phase (lag phase—the time between influx and efflux of Ca^2+^). We observed an acceleration of the mPTP opening in the Ethanol RLM compared to the Control RLM (Figure 1a vs. Figure 1c). The addition of 1 μM of Cbx results in the induction of the mPTP opening in the Control and the Ethanol RLM (Figure 1a vs. Figure 1b and Figure 1c vs. Figure 1d, respectively). Moreover, we found that the addition of Cbx had a stronger effect on Ca^2+^ fluxes and mitochondrial ΔΨm changes in rats with alcohol intoxication. Quantitative characteristics of these effects are shown in Figure 2. The diagram in panel (a) shows quantitative changes in the rates of Ca^2+^ influx (green columns) and Ca^2+^-induced dissipation of membrane potential ΔΨm (red columns) calculated as the TPP^+^ influx rate (V^TPP+^_in_, nmol min^−1^ mg^−1^ of protein). As can be seen in the figure, the addition of Cbx leads to a decrease in the influx rates of Ca^2+^ and TPP^+^, which reflects the facilitation of the mPTP induction. Thus, in the presence of Cbx, VCa^2+^_in_ decreases by 11% and V^TPP+^_in_—by 23% in Control RLM (Columns 2 vs. 1). However, in the mitochondria of Ethanol rats, the effect of Cbx increases to 33% and 60% dotted columns 4 vs. 3), respectively, which indicates an increase in the effect of Cbx in RLM under alcohol intoxication. In addition, it should be noted that in Ethanol RLM influx rates are lower than in Control RLM (Columns 3 vs. 1). Panel (b) (green columns) shows data on quantitative changes in the calcium retention capacity (CRC, concentration of Ca^2+^ added to the mitochondrial suspension in which the Ca^2+^ ions accumulating in the mitochondria induce the mPTP to open) of RLM upon mPTP opening. Cbx has been shown to have little effect on this parameter in the Control RLM (by about 15% (green bar 2 vs. 1) and doubles its effect in Ethanol RLM by 30% (green columns 4 vs. 3). The changes in the red columns on the diagram on the Figure 2b showing values of the lag phase (the time between the influx and efflux of Ca^2+^ during the last Ca^2+^ addition) generally correlate with the changes in the CRC, thereby confirming the effect of Cbx on the Ca^2+^ flows in both Control and Ethanol RLM. Thus, the addition of Cbx to the Control RLM practically does not affect the value of the lag phase (red column 2 vs. 1), while in Ethanol RLM, lag phase decreases almost to zero (10 times compared to the lag phase without additions, red columns 4 vs. 3).

**Figure 3:**
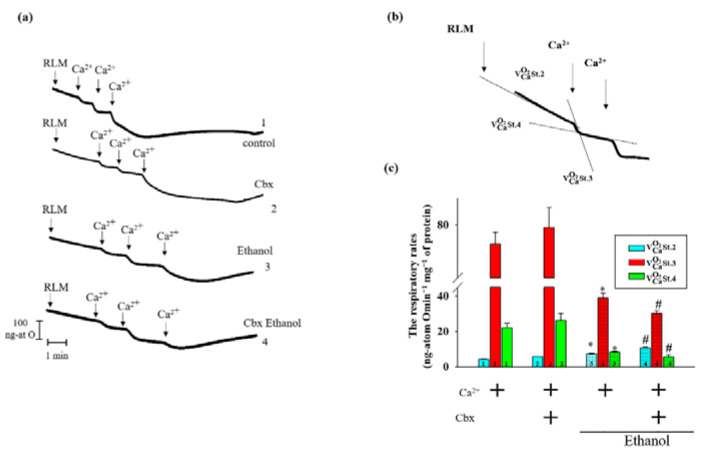
Effect of Cbx on respiratory activity in RLM. The conditions were the same as in Figure 1. (**a**) Representative curves of respiratory activities. (**b**) A schema of calculation of oxygen consumption rates in states 2–4 ($V_{\mathrm{Ca}}^{O_{2}}\mathrm{St}.2$, $V_{\mathrm{Ca}}^{O_{2}}\mathrm{St}.3$ and $V_{\mathrm{Ca}}^{O_{2}}\mathrm{St}.4$). (**c**) Quantitative analysis of respiratory activity in Control and Ethanol RLM in the presence/absence of Cbx. The values shown are the means ± SD from three independent experiments; * *p* ≤ 0.05 compared with the control value in the RLM without additions (columns 1); # *p* ≤ 0.05 compared with the control value in the Ethanol RLM without additions (dotted columns 3).

Under the same conditions, the respiratory activities of the RLM were measured (Figure 3). On the Panel (a) the curves of mitochondrial respiration in experimental conditions are shown. Panel (b) is schematically represented a model for the calculation of oxygen consumption rates in state 2 (Substrate-dependent phosphorylation, respiration rate before Ca^2+^ addition; $V_{\mathrm{Ca}}^{O_{2}}\mathrm{St}.2$; ng-atom O min^−1^ mg^−1^ of protein), state 3 (Maximum respiration rate stimulated by Ca^2+^ addition; $V_{\mathrm{Ca}}^{O_{2}}\mathrm{St}.3$; ng-atom O min^−1^ mg^−1^ of protein) and state 4 (respiratory rate at rest after a Ca^2+^ accumulation);$\;V_{\mathrm{Ca}}^{O_{2}}\mathrm{St}.4$; ng-atom Omin^−1^ mg^−1^ protein). The evaluation of these parameters is presented in Figure 3c. Primarily, we did not observe any significant changes in the rates of mitochondrial respiration when Cbx was added to the Control RLM (all columns 2 vs. 1). However, in Ethanol RLM the rate of substrate-dependent respiration (state 2) increases in the presence of Cbx by 40% (blue columns 4 vs. 3), while the respiration rate in state 3 and the rate of oxygen consumption in state 4 were significantly decreased (green and red columns 4 vs. 3). The rates of mitochondrial respiration in Ethanol RLM also changed in comparison with the Control in a similar manner. Thus, $V_{\mathrm{Ca}}^{O_{2}}\mathrm{St}.2$ enhances and $V_{\mathrm{Ca}}^{O_{2}}\mathrm{St}.3$ and $V_{\mathrm{Ca}}^{O_{2}}\mathrm{St}.4$ reduce in Ethanol RLM compared to Control. This indicates a greater sensitivity to Ca^2+^-overloading and mPTP initiation in the Ethanol RLM.

**Figure 4:**
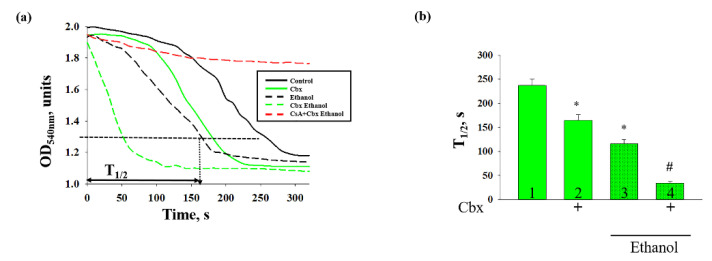
Effect of Cbx on the Ca^2+^-induced swelling of the Control and Ethanol RLM. (**a**) Representative curves of RLM swelling. (**b**) Average half-times (T_1/2_) of swelling. The values shown are the means ± SD from three independent experiments; * *p* ≤ 0.05 compared with the control value in the RLM without additions (columns 1); # *p* ≤ 0.05 compared with the control value in the Ethanol RLM without additions (dotted columns 3).

The addition of Ca^2+^ at the threshold concentration to the mitochondrial suspension incubated in the standard medium described in Section 4 caused a decrease in light scattering, which is indicator of mitochondrial swelling. It is considered one of the most important characteristics of mPTP. At the next step, the swelling of the Control and Ethanol RLM in the absence/presence of Cbx was measured. Figure 4a indicates the curves of mitochondrial swelling of RLM. We observed that the addition of Cbx leads to the stimulation of swelling in both Control and Ethanol RLM; however, this effect is more pronounced in the mitochondria isolated from rats with alcohol intoxication. It should be noted that Ethanol RLM is more sensitive to calcium therefore mitochondrial swelling is accelerated in comparison with the Control RLM. The quantitative characteristics of the observed effects are presented in Figure 4b. The swelling process was characterized by the time needed to reach the half-maximal light scattering signal (T_1/2_). Thus, in the Ethanol RLM, T_1/2_ was reduced twice compared to Control RLM, indicating a greater sensitivity of mitochondria to Ca^2+^ and mPTP initiation in alcohol intoxication. Simultaneously, the effect of Cbx on mitochondrial swelling in Ethanol RLM was two times stronger than in Control one (35%, Columns 2 vs. 1 in Control RLM and 70%, columns 4 vs. 3 in Ethanol RLM, respectively). It should be noted that these effects were cyclosporin A (CsA)-sensitive, confirming the association of the observed phenomena with mPTP.

**Figure 5:**
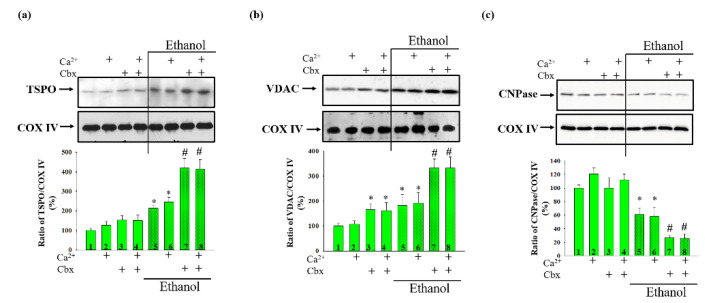
Effect of Cbx on the level of mitochondrial proteins—regulators of mPTP: TSPO—(**a**), VDAC—(**b**) and CNPase—(**c**) The upper parts represent Western blots stained with the corresponding antibodies. Lower parts—quantitation of immunostaining using computer-assisted densitometry. The protein band intensity was quantified after normalization to COX IV. The values shown are the means ± SD from three independent experiments. The level of each protein in the Control RLM without additions was taken as 100%. * *p* ≤ 0.05 compared with the corresponding value in the Control RLM without Cbx additions with opened and closed mPTP, respectively (Columns 3, 5 vs. column 1 for the mPTP closed state and columns 4, 6 vs. 2 for the mPTP opened state), # *p* ≤ 0.05 compared with the corresponding value in the Ethanol RLM without Cbx additions (dotted columns 7 vs. 5 in closed mPTP and 8 vs. 6 in opened mPTP).

Next, we tested the effect of Cbx on the content of mitochondrial proteins, which are considered regulators of mPTP (Figure 5). The levels of translocator protein (TSPO), voltage dependent anion channel (VDAC) and 2′,3′-cyclic nucleotide-3′-phosphodiesterase (CNPase) in the Control and Ethanol RLM were investigated. The changes in content of the studied proteins were determined using Western blot, samples for analysis were taken from the incubation chamber under conditions of opened/closed mPTP in the absence/presence of Cbx.

The results presented in Figure 5a show the absence of the effect of Cbx on the content of TSPO in the mitochondria of Control rats, in both closed and opened mPTP (columns 3 vs. 1 and 4 vs. 2, respectively). However, in Ethanol RLM, Cbx increases the level of TSPO almost two times in both the closed (columns 7 vs. 5) and opened mPTP (columns 8 vs. 6). Next, we analyzed the change in the VDAC content under our experimental conditions. As shown in Figure 5b, Cbx increased the level of VDAC in RLM isolated from Control and Ethanol rats. However, in Ethanol RLM the effect of Cbx is more pronounced. Thus, in Control RLM, Cbx enhances the level of VDAC by 60% in both the closed (columns 3 vs. 1) and opened mPTP (columns 4 vs. 2), and in Ethanol RLM the increasing effect was about 80% (Columns 7 vs. 5 and 8 vs. 6, respectively).

Finally, we analyzed the content of CNPase, another regulator of mPTP. In the lower part of Figure 5c, a diagram reflecting changes in the level of CNPase, is presented. The results show the absence of the effect of Cbx on the content of CNPase in the mitochondria of Control rats in both the closed and opened mPTP (columns 3 vs. 1 and 4 vs. 2, respectively), as in the case of TSPO. In contrast to the Control RLM, in Ethanol Cbx affects the CNPase level, reducing it by more than three times when mPTP was closed (columns 7 vs. 5) and opened (columns 8 vs. 6).

**Figure 6:**
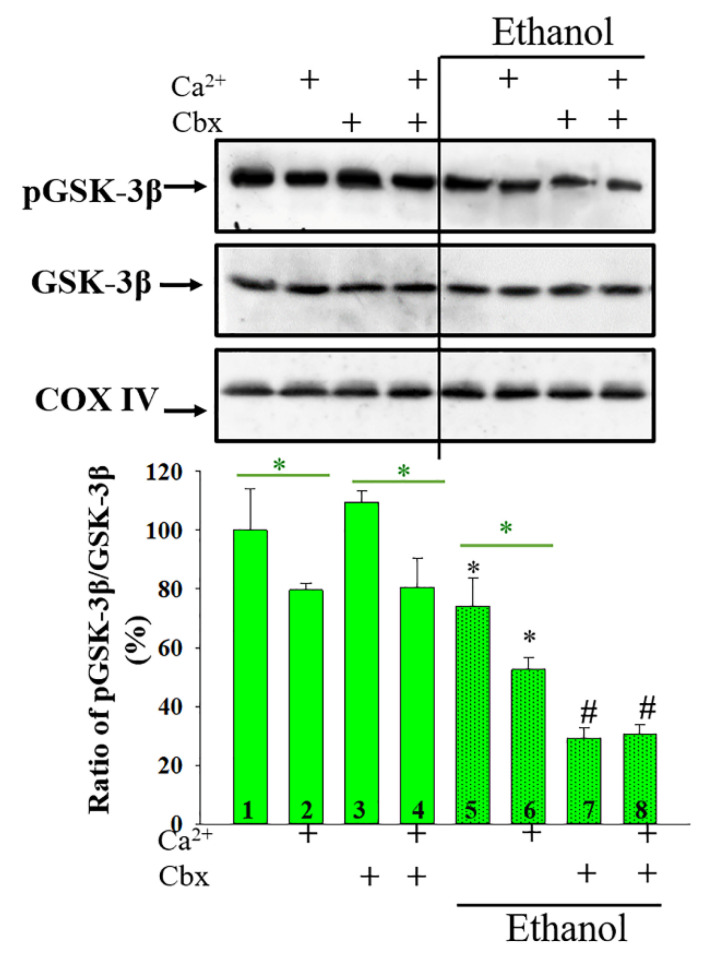
The phosphorylation states of stress-activated protein kinase GSK3β in RLM. Upper parts represent Western blots of a mitochondrial suspension after the isolation of RLM (see Section 4). Lower parts—quantitation of immunostaining using computer-assisted densitometry. Bar graphs represent the ratios of pGSK3β to total GSK3β in the Control and Ethanol RLM. The protein band intensity was quantified after normalization with respect to COX IV. The values shown are the means ± SD from three independent experiments; The ratios of pGSK3β to total GSK3β in the Control RLM without additions was taken as 100%. * *p* ≤ 0.05 compared with the corresponding value in the Control RLM without Cbx additions with opened and closed mPTP, respectively (Columns 3, 5 vs. column 1 for the mPTP closed state and columns 4, 6 vs. 2 for the mPTP opened state), # *p* ≤ 0.05 compared with the corresponding value in the Ethanol RLM without Cbx additions (dotted columns 7 vs. 5 in closed mPTP and 8 vs. 6 in opened mPTP).

It is widely known that the protein kinases participate in mitochondrial signaling pathways. These pathways include changes in the functioning of mPTP and lead to the induction of programmed cell death [22]. It was previously shown that many molecular processes in mitochondria that occur in various pathologies are regulated by phosphorylation/dephosphorylation of glycogen synthase kinase 3 (GSK-3β) [23]. Here, we analyzed alterations in the ratio of the phospho-GSK3β-to-total GSK3β in RLM under conditions of chronic alcohol intoxication in comparison with control conditions and investigated the possible effect of Cbx on these alterations. The results are presented in Figure 6. We found that the opening of mPTP inhibits GSK-3β phosphorylation, leading to its activation by 20% in both Control (columns 2 vs. 1) and Ethanol (columns 6 vs. 5) RLM.

The presence of Cbx did not change the level of GSK-3β in Control RLM when mPTP was closed (columns 3 vs. 1) and opened (columns 4 vs. 2). In Ethanol RLM the addition of Cbx caused a decrease of GSK-3β phosphorylation in both the closed and opened mPTP by more than two times and 40% (Columns 7 vs. 5 and 8 vs. 6, respectively). However, it should also be noted that, in the presence of Cbx in Ethanol RLM, the level of GSK-3β phosphorylation did not change with the opening of mPTP (Columns 8 vs. 7).

## 3. Discussion

An increase in the permeability of mitochondrial membranes is the cause of multiple disturbances in the functioning of mitochondria, leading to cell death both along the pathway of apoptosis and necrosis [24,25,26]. One of the main pathways for the development of changes in the permeability of mitochondrial membranes is the opening of mPTP in response to stress or an increase the threshold Ca^2+^ concentration. The opening of mPTP leads to mitochondrial depolarization, the drop of ΔΨm, the enhanced of ROS production, impaired cellular Ca^2+^ homeostasis, mitochondrial swelling and release of pro-apoptotic factors into the cytosol to initiate cell death [27,28]. Thus, mPTP is a key factor in the development of various diseases caused by mitochondrial pathologies. In particular, there is evidence that alcohol intoxication results in disturbances in the functioning of the mPTP [8,10]. Chronic alcohol consumption increases the sensitivity of mitochondria to the induction of mPTP and, as a result, enhances the permeability of the inner mitochondrial membrane, as well as its depolarization, swelling and causes a damage to the outer membrane [7,11]. However, the mechanism of action of ethanol on mitochondria remains unclear. In addition, despite the long-term and intensive study, the composition and structure of the mPTP have not yet been established. Therefore, the search for possible regulators of mPTP, as well as targets for potential therapeutic effects on its components in the treatment of various pathologies is continued.

It should be noted that we have previously shown that Cbx was an effective inducer of mPTP in the mitochondria of the rat brain and liver [18]. Here, we found that Cbx enhanced its effect on the main parameters of mPTP functioning in Ethanol RLM (Figure 2). In both Control and Ethanol RLM, Cbx reduced the rates of Ca^2+^ and TPP^+^ influx (which reflects a decrease in the membrane potential) (Figure 2a), as well as the calcium retention capacity (CRC) and lag phase (Figure 2b). However, its effect on these parameters of mPTP was stronger in Ethanol RLM than in Control RLM. Moreover, Cbx had a similar effect on the swelling of Ethanol RLM (Figure 4). Thus, in Ethanol RLM, it accelerated swelling two times stronger than in Control.

We have previously shown that two other pharmacological agents, Protoporphyrin IX and PK 11195, have the same enhancing effect in Ethanol RLM [11]. Protoporphyrin IX and PK 11195 are ligands of the TSPO but they are also capable of acting by other, TSPO-independent mechanisms [29]. Our data show that the effect of Cbx on mPTP functioning can also be mediated through TSPO [18]. We hypothesized that long-term exposure to alcohol in rat liver mitochondria develops a special mechanism of mPTP functioning, which differs from the mechanism acting under control conditions. Apparently, one of the potential regulators of this mechanism can be Cbx. It should also be noted that Ethanol RLM are generally more sensitive to the induction of mPTP, they have a reduced CRC, lag phase and an increased rate of swelling compared to Control RLM that is consistent with our previous data and the results of other researchers [11,30,31]. We also observed changes in respiratory activities after Ca^2+^ addition to Ethanol RLM compared to Control one. At the same time, Cbx had no effect on the parameters of respiratory activities in Control RLM, while in Ethanol RLM it decreased the respiration rate (state 3) and increased the rate of substrate-dependent respiration (state 2) (Figure 3). Together, these changes indicate an “uncoupling” of the energy-transforming systems in the inner mitochondrial membrane and an initiation of mPTP opening. These data suppose the special role of Cbx in mitochondria in chronic alcohol intoxication.

In order to confirm the participation of Cbx in ethanol-induced enhancement of mPTP induction, as well as to identify the participants of this mechanism, in our work the effect of Cbx on changes in the levels of regulator proteins of mPTP in mitochondria of Control and Ethanol RLM was investigated.

The TSPO is considered to be one of the key proteins that regulate the functioning of mPTP [32,33]. It is known that TSPO is directly involved in the regulation of mPTP in various pathologies [11,34,35,36]. We have recently shown an increase in its level, as well as the involvement of its ligands in the regulation of mPTP in RLM with chronic alcohol intoxication [11]. Here, we also found an increase in TSPO content in Ethanol RLM compared to Control when the mPTP was opened or closed. It should be noted that increased content of TSPO has been observed in a wide variety of cancer, including brain cancers [37,38], breast cancers [39,40], prostate cancers [41], colon cancers [42,43] and hepatic carcinomas [44] and, therefore, it is a biomarker in therapeutic diagnosis [45,46]. We suppose that in diseases associated with mitochondria, TSPO also accumulates in comparison with normal conditions, and can serve as a biomarker in the diagnosis of mitochondrial pathologies. Cbx does not affect the level of TSPO in Control RLM, however, Cbx significantly enhances the level of TSPO in Ethanol RLM. Apparently, under normal conditions, Cbx has other targets of action in the mitochondria. These data are in good agreement with the data obtained from measurements of the most important parameters of the mitochondria. Since the research of our group, as well as literature data, indicate a tight relationship between TSPO and VDAC [32,47,48], we also investigated the effect of Cbx on VDAC level in the mitochondria of control animals and animals with chronic alcohol dependence. We have shown that Cbx in Ethanol RLM also increases the content of VDAC compared to Control RLM when the mPTP was opened or closed. The role of VDAC in the functioning of mPTP is controversial and has not yet been clarified. VDAC was originally thought to be a structural component of a pore; later experiments with knockout mice showed that knockout of all three VDAC isoforms did not interfere with the functioning of mPTP [49]. However, at present there is a large number of works reporting new evidence for the involvement of VDAC in mPTP [50,51,52,53]. Apparently, such discrepancies are associated with the fact that the role of VDAC in mitochondria and its participation in mPTP varies depending on conditions, for example, in health and in various pathologies. Earlier, we showed that VDAC in mitochondria co-localized with another mPTP regulator such as 2′,3′-cyclic nucleotide 3′-phosphodiesterase (CNPase) [54]. CNPase is myelin sheath protein found by us in the mitochondria of unmyelinated tissues, which is also involved in the functioning of mPTP [55]. In this work, it was shown that Cbx is not able to affect the level of CNPase (as well as TSPO) in Control RLM and, on the contrary, it decreases the content of CNPase in Ethanol RLM (the opposite effect compared to TSPO). Thus, we found an inverse correlation between the content of TSPO and VDAC, on the one hand, and CNPase, on the other, under the influence of Cbx in the liver mitochondria of rats with chronic alcohol intoxication. It should be noted that we have previously observed a similar inverse correlation between the levels of these proteins in Ethanol RLM under the influence of TSPO ligands [11]. Obviously, a decrease in the level of CNPase in pathological conditions leads to an increase in the content of its substrates, 2′,3′-cAMP and 2′,3′-cNADP, the deleterious effect of which results in the initiation of the mPTP. The alteration in the level of CNPase causes an increase in the level of CNPase’s partner proteins in the mitochondria and can be increased by the influence of Cbx.

The obtained data suggest that under pathological conditions of chronic alcohol exposure, changes in both the structure of mPTP and the mechanisms of its regulation can occur in the mitochondria. These alterations can be a compensatory response to the damage that occurs during intoxication. We hypothesize a functional interaction between TSPO, CNPase and VDAC in mitochondria in response to ethanol-induced degenerative changes. Additional research is needed to clarify the mechanism of this interaction and its functional significance. However, our studies show that these proteins are potential targets for therapeutic regulation. The enhanced effect of Cbx on the components of the system indicates its involvement in regulatory mechanisms under conditions of the development of alcohol intoxication.

It is known that most of the biological functions of mitochondria, similar to other cellular organelles, are modulated by phosphorylation of protein kinases [56,57,58,59]. Many of mitochondrial membrane-bound proteins, are phosphorylated by glycogen synthase kinase 3 (GSK-3β), in particular, VDAC [60,61], cyclophilin D and translocase of adenine nucleotides [62,63,64]. The role of GSK-3β in determining the threshold for mPTP opening was observed [65,66]. It was shown also that the ratio of pGSK-3β/GSK-3β correlates with the threshold Ca^2+^ concentration needed to induce mPTP opening in mitochondria from rat cardiomyocytes [67]. GSK-3β is phosphorylated by a serine/threonine protein kinase, Akt [68], protein kinase A or ribosomal s6 kinase [69,70]. The phosphorylation of GSK-3β leads to its inactivation [71]. Here we have shown that the opening of mPTP results in dephosphorylation and, consequently, the activation of GSK-3β in both Control and Ethanol RLM, which is in good agreement with the literature data (Figure 6). We also found a significant decrease in the level of phosphorylation of GSK-3β in Ethanol RLM in comparison with the Control RLM. Cbx had no effect on the phosphorylation status of GSK-3β in Control RLM; however, it significantly reduced this parameter in Ethanol RLM. Thus, we assume that the system that occurs during chronic alcohol intoxication in the mitochondria can be regulated by protein kinases, in particular GSK-3β, which, in turn, activates/inactivates mitochondrial proteins by phosphorylation/dephosphorylation.

## 4. Materials and Methods

### 4.1. Animals and Treatment

To study the effects of alcohol on mitochondrial functions, model experiments were performed on rats. In our experiments, we used the Lieber–DeCarly model of chronic alcohol intoxication, which allows one to achieve the consumption of alcohol at high doses [72]. Mixtures for preparing liquid nutrition were manufactured by BioServ (Frenchtown, NJ, USA). The control diet contained fats, proteins, carbohydrates, trace elements and vitamins, with 18% of the total calories coming from proteins, 35% from fats and 47% from carbohydrates. In the alcohol diet, 36% of the calories from the carbohydrate components were replaced by calories from ethanol, the concentration of which was 5% in the final diet. This model used isocaloric pair-feeding of the animals. For this process, male rats of the same age and weight, divided into pairs, were kept in separate cages equipped with special graduated drinking bowls, without access to water and solid food. Eight rats (aged two months) were used in the experiments, with four in each group. Rats that received an alcoholic diet had free access to food throughout the day, and the control rats received an amount of food equivalent to that consumed by their paired alcoholic rats; food consumption was measured daily. Over a 10-day period of habituation, the rats received a gradually increasing amount of ethanol (0, 1, 2, 3, 4 and 5%) in their food, and then all alcoholic rats received food containing 5% ethanol for 8 weeks. At the start of the experiment, the average weight of the rats was 167.87; during the experiment, the weight gain was 163.12 ± 11.55 g. The rats consumed an average of 60–80 calories daily, and alcoholic rats received 14.86 ± 16.25 g of ethanol per 1 kg rat weight, which is consistent with the published data.

The complete data of the body mass of each animal before and after treatment, as well as the weights of the livers and average values, are presented in Table 1.

All experiments were performed in accordance with the Regulations for Studies with Experimental Animals (Decree of the Russian Ministry of Health of 12 August 1997, No.755). The protocol was approved by the Commission on Biological Safety and Ethics at the Institute of Theoretical and Experimental Biophysics, Russian Academy of Sciences (February 2021, protocol N18/2021).

### 4.2. Isolation of Rat Liver Mitochondria

Rat liver mitochondria (RLM) were isolated from rats by the standard method using a homogenization medium containing 210 mM mannitol, 70 mM sucrose, 1 mM EGTA, 0.05% bovine serum albumin fraction V, and 10 mM Tris (pH 7.3). The homogenate was centrifuged at 800× *g* for 10 min to pellet the nuclei and damaged cells. The supernatant containing the mitochondria was centrifuged for 10 min at 9000× *g*. Sedimented mitochondria were washed twice in a medium without EGTA and BSA for 10 min at 9000× *g* and resuspended in the same medium. The protein concentration was then determined using a Bradford assay.

Mitochondria (1 mg protein/mL) were incubated at 25 °C in a medium containing 125 mM KCl, 10 mM Tris (pH 7.4) and 2 mM K_2_HPO_4_. In the experiments, glutamate (5 mM) and malate (5 mM) were used as respiratory substrates. The oxygen consumption rate was measured with a Clark-type O_2_ electrode that was integrated into a 1-mL multifunctional chamber for the simultaneous registration of Ca^2+^ flux and respiratory activity. All experiments were performed in an opened chamber.

The Ca^2+^ and TPP^+^ flows of RLM were determined with TPP^+^- and Ca^2+^-sensitive electrodes (Nico, Russia) in a 1 mL measuring chamber [59]. The mitochondria (0.5 mg protein/mL) were incubated at 25 °C in a medium containing 125 mM KCl, 10 mM Tris (pH 7.4) and 2 mM K_2_HPO_4_; glutamate (5 mM) and malate (5 mM) were used as substrates. The mPTP opening in RLM was induced by a threshold Ca^2+^ concentration (the first addition of Ca^2+^ contained 50 nmol Ca^2+^ per mg of protein, while the second and third contained 90 nmol Ca^2+^ per mg of protein). The parameters of Ca^2+^ transport, such as the calcium retention capacity (V^Ca2+^_in_—the rate of Ca^2+^ release from mitochondria, CRC—concentration of Ca^2+^ added to the mitochondrial suspension in which the Ca^2+^ ions accumulating in the mitochondria induce the mPTP to open) and the lag phase (the time between influx and efflux, s) were measured. The Ca^2+^-induced dissipation of the membrane potential was measured as the TPP^+^ infllux rate (V^TPP+^_in_, nmol min^−1^ mg^−1^ of protein). To study the influence of the Cbx, it was pre-incubated with the mitochondria for 10 min at a concentration of 1 μM.

The swelling of the RLM was measured by changes in the light scattering in the mitochondrial suspension at 540 nm (A540) and 25 °C using a Tecan I-Control infinite 200 spectrophotometer. The standard incubation medium for the swelling assay contained 125 mM KCl, 10 mM Tris (pH 7.4), 2 mM KH_2_PO_4_ and 5 mM glutamate, with 5 mM malate as a substrate. The concentration of the mitochondrial protein in each well was 1 mg protein/mL. Swelling was initiated via the addition of 190 nmol of Ca^2+^ per mg of protein. The swelling process was characterized by the time needed to reach the half-maximal light scattering signal (T1/2). The top and the bottom plateau for half-maximal light scaterring signal(A/2) was assigned for each curve individually using SigmaPlot functions.

### 4.3. Electrophoresis and Immunoblotting of the Mitochondrial Proteins

Aliquots of mitochondria in the incubated medium from the chamber (100 μL) were taken from the chamber, placed in an Eppendorf tube and centrifuged for 3 min at 15,000× *g*. The sediments were lysed with a lysis buffer (50 mM Tris–HCl (pH 7.4), 150 mM NaCl, 1% Triton X-100, 0.1% SDS, 1 mM EDTA, 1 mM Na_3_VO_4_ and 1 mM NaF) supplemented with proteinase/phosphatase inhibitors. Then, the obtained samples were solubilized in Laemmli buffer (Bio-Rad, Hercules, CA, USA). Samples were heated to 95 °C for 5 min. Next, 20 μg of each sample was applied to the gel and subjected to electrophoresis under denaturing conditions by 12.5% SDS-PAGE and transferred to a nitrocellulose membrane for the following Western blot analysis. Precision Plus Pre-stained Standards from Bio-Rad Laboratories (Hercules, CA, USA) were used as markers. After 1 h blocking, the membrane was incubated with the appropriate primary antibody. The Polyclonal TSPO antibody (1:1000), were obtained from Abcam, Cat. # ab 108489 (Cambridge, UK); the monoclonal VDAC antibody (1:1000) was purchased from Calbiochem, Cat. #PC548. (San Diego, CA, USA); the polyclonal rabbit phospho-GSK-3β (Ser9) were obtained from Cell Signaling, Cat. # 9336S (Leiden, The Netherlands). The monoclonal anti-CNP antibody (anti-CNP Ab) was obtained as described [73] and used at a 1:10,000 dilution, the monoclonal GSK3β antibody (1:2000) were obtained from Abnova, Cat. #Mab 10672 (Taipei, Taiwan). The monoclonal COX IV antibody (Abcam, Cat. #ab14744) was used as a loading control. Immunoreactivity was detected using the appropriate secondary antibody conjugated with horseradish peroxidase (Jackson Immuno Research, West Grove, PA, USA). Peroxidase activity was detected using ECL chemiluminescence reagents (Bio-Rad, Hercules, CA, USA).

### 4.4. Statistical Analysis

For the statistical analysis, the relative levels of protein density were expressed as the mean ± SD from at least three to four independent experiments. The statistical significance of the difference between the mean values was evaluated using Student’s *t*-test. A difference was considered significant at *p* < 0.05.

## 5. Conclusions

In summary, we found that the effect of Cbx significantly enhanced in the liver mitochondria of rats with chronic alcohol intoxication related to Control RLM. This effect was pronounced in a decrease in the lag phase, acceleration of Ca^2+^ release from mitochondria and Ca^2+^ -induced mitochondrial swelling, as well as changes in the level of mitochondrial proteins involved in the regulation of mitochondrial membrane permeability. Thus, we concluded that carbenoxolone is involved in the regulation of the functioning of mPTP in liver mitochondria in alcoholic pathology. The mechanism of this action remains the subject of further research. According to modern concepts, mPTP does not have constant structural components and in different conditions, various protein components and channels can participate in its formation. In conditions of chronic alcohol intoxication, other protein components may take part in the formation of mPTP than under normal conditions. Therefore, under conditions of chronic alcohol intoxication, mPTP can be formed with the participation of VDAC, CNPase and TSPO, one of which can be a target of carbenoxolone. The obtained data allow us to become closer to understanding the pathological processes occurring during chronic alcohol intoxication in the liver mitochondria and provide additional information about the possible side effects of the simultaneous use of carbenoxolone and alcohol.

## Figures and Tables

**Table 1:** Changes in weight characteristics after the ethanol treatment of rats.

№, Pair	Control				№, Pair	Ethanol			
	Body Weight, g		Liver Weight Aft. Exp, g	Ratio of Body/Liver weight Aft. Exp, %		Body Weight, g		Liver Weight aft. Exp, g	Ratio of Body/Liver Weight Aft. Exp, %
	Bef. Exp.	Aft. Exp.				Bef. Exp.	Aft. Exp.		
1 (C1)	155	337	10.4	3.08	1 (E1)	157	312	11.5	3.08
2 (C2)	161	342	10.2	2.99	2 (E2)	160	326	10.9	2.99
3 (C3)	164	348	10.5	3.02	3 (E3)	167	315	12.1	3.02
4 (C4)	158	337	10.7	3.17	4 (E4)	155	314	11.7	3.17
mean	159.5 ± 3.9	341 ± 5.2	10.45 ± 0.2	3.06 ± 0.08	mean	159.7 ± 5.3	316.7 ± 6.3	11.5 ± 0.5	3.64 ± 0.2

## Data Availability

The data presented in this study are contained within this article.
